# Intravenous Immunoglobulin Combined With Corticosteroids for the Treatment of Stevens–Johnson Syndrome/Toxic Epidermal Necrolysis: A Propensity-Matched Retrospective Study in China

**DOI:** 10.3389/fphar.2021.750173

**Published:** 2022-01-18

**Authors:** Lu Yang, Yan-Hong Shou, Feng Li, Xiao-Hua Zhu, Yong-Sheng Yang, Jin-Hua Xu

**Affiliations:** Department of Dermatology, Huashan Hospital, Fudan University, Shanghai, China

**Keywords:** Stevens–Johnson syndrome (SJS), toxic epidermal necrolysis (TEN), corticosteroid, intravenous immunoglobulin (IVIg), TEN-specific severity-of-illness score (SCORTEN), propensity score matching (PSM)

## Abstract

**Background:** Stevens–Johnson syndrome (SJS) and toxic epidermal necrolysis (TEN) are rare but life-threatening severe adverse drug reactions. The use of corticosteroids and intravenous immunoglobulin (IVIg) in SJS/TEN remains controversial.

**Methods:** In this single-center, observational, propensity-matched, retrospective study, we collected a total of 224 patients with SJS/TEN who were hospitalized in our department from 2008 to 2019; according to treatment with IVIg combined with corticosteroids or with corticosteroids alone, patients were divided into combination therapeutic group (163 patients) and monotherapeutic group (61 patients). Patients from the two groups were matched by their propensity score in blocks of 2:1. Comparisons of the clinical characteristics and prognoses between propensity-matched SJS/TEN patients treated with IVIg combined with corticosteroids and corticosteroids alone were made.

**Results:** After our propensity matching, a total of 145 patients were yielded, including 93 patients treated with IVIg and 52 patients not treated with IVIg. All of the 23 variables reflected good matching between patients treated with/without IVIg, and no significant difference was observed. Although there was no significant difference between the totally predicted and actual mortality in both of our groups, the actual mortality was lower than it was predicted in patients treated with IVIg [*p* > 0.250, the standardized mortality ratio (SMR) was 0.38, 95% CI 0.00–0.91] and patients treated without IVIg (*p* = 1.000, the SMR was 0.75, 95% CI 0.00–1.76). IVIg tended toward reducing the time to arrest of progression by 1.56 days (*p* = 0.000) and the length of hospital stay by 3.37 days (*p* = 0.000). The mortality rate was 45% lower for patients treated with IVIg combined with corticosteroids than those only treated with corticosteroid therapy, although it was not statistically significant (*p* = 0.555). The incidence of skin infections was significantly lower in the combined therapy group (*p* < 0.025), and the total infection rate of patients treated with combination therapy tended to decrease by 67% compared to patients treated with corticosteroids alone (*p* = 0.047).

**Conclusion:** The actual mortality rate of patients treated with corticosteroids alone or IVIg combined with corticosteroids tended to be lower than those predicted by TEN-specific severity-of-illness score (SCORTEN), although there was no significance. Compared with those treated by corticosteroids alone, combination therapy was prone to bring a better prognosis for SJS/TEN patients.

## 1 Introduction

Stevens–Johnson syndrome (SJS)/toxic epidermal necrolysis (TEN) is a rare but life-threatening spectrum of drug-related adverse reactions manifesting as blisters and erosions, which always involve the skin, mucous membranes, and several internal organs. SJS/TEN is classified according to the extent of skin involvement. SJS involves less than 10% of total body surface area (TBSA), whereas SJS/TEN overlap involves 10%–30% TBSA, and TEN is defined as detachment of more than 30% TBSA. As the primary adverse outcome, the mortality in patients with SJS/TEN is always predicted by TEN-specific severity-of-illness score (SCORTEN), which was developed and validated by Bastuji-Garin et al. (2000) in 2000. Age above 40 years, initial percentage of epidermal detachment above 10%, malignancy, serum glucose above 14 mmol/L, tachycardia above 120/min, bicarbonate below 20 mmol/L, and serum urea above 10 mmol/L were assigned one point each; the higher the score, the higher the predicted mortality risk.

During the last few years, standard medical therapy for SJS/TEN remains controversial, except supportive care is widely recommended, and the benefits of other therapies have not been entirely approved currently, especially in terms of the use of systemic corticosteroids and intravenous immunoglobulin (IVIg) (White et al., 2018). In the past few decades, systematic corticosteroids were generally administered in our SJS/TEN patients. However, the combination therapy with systemic corticosteroids and IVIg has begun to be applied in our department since 2001 and have increasingly been used in China (Y Yang et al., 2009). In situations in which clinical trials are difficult to perform because of the rarity and severity of the disease, we decided to conduct a retrospective study in patients treated with or without IVIg in our department from 2008 to 2019 when confounding variables were controlled with a propensity-matched logistic regression in order to evaluate the curative effect of IVIg objectively.

## 2 Materials and Methods

### 2.1 Patients

We performed a retrospective study from January 2008 to December 2019 to examine all patients with integrated medical records admitted to the intensive care unit of the Department of Dermatology of Huashan Hospital affiliated with Fudan University diagnosed with SJS/TEN. The inclusion criteria were as follows: 1) SJS/TEN was diagnosed by the same group of doctors according to clinical data and skin biopsy (all patients with complicated conditions underwent skin biopsy pathologically showing full-thickness necrosis of the epidermis and a sparse dermal inflammatory infiltrate). 2) Disease progressed within the 24 h preceding admission. 3) Medical records were integrated. Patients who were diagnosed with staphylococcal scalded skin syndrome (SSSS), autoimmune blistering diseases, erythema exsudativum multiforme majus (EEM), or other types of severe drug eruptions were excluded. A total number of 224 patients were included in this study, and informed consent was obtained from all patients. According to treated with or without IVIg, they were divided into two groups. All patients received systemic corticosteroids (methylprednisolone with an initial dose in the range 1–1.5 mg/kg/day or equivalent hydrocortisone/dexamethasone) as early as possible and symptomatic supportive treatments consisting of wound care, pain relief, fluid compensation, nutritional assessment, and electrolyte balancing. If the patient’s condition continued to progress within 48 h after admission, a total dose of 2 g/kg of IVIg (dose of 0.4 g/kg/day of IVIg for 5 days) combined with corticosteroid would be performed for patients with normal renal function. In our study, 163 patients received combination therapy. This study was approved by the Ethics Committee of Fudan University, Affiliated Huashan Hospital, Shanghai, China.

### 2.2 Data Collection

Patient characteristics included relevant demographics, TBSA, presence of mucous membrane involvement, vital signs, laboratory data, physical examination, SCORTEN score, causative agents, suspected drug allergy history, underlying diseases, timing from disease onset to admission, time to the arrest of progression, length of hospital stay, observed mortality, corticosteroid treatment modalities and dosages, and associated complication infection, which was diagnosed by a positive culture combined with clinical evidence, including local pain symptoms and unexplained fever. Specific criteria of infection in different sites were characterized as described previously (L Yang et al., 2020). Data were collected independently by 2 investigators, LY and Y-HS. Medical information was cross-checked by Y-SY and J-HX.

### 2.3 Propensity Matching

Propensity score matching (PSM) is a method that controls confounders between treatment and control groups and reduce the impact of confounding factors on the therapeutic effect estimation in observational studies (Rubin and Thomas, 1996). In our study, patients treated by IVIg combined with corticosteroids were classified into the treatment group, and patients treated with corticosteroids alone were placed in the control group for propensity matching. A propensity score was derived from a non-parsimonious logistic regression model on the basis of baseline characteristics: age, gender, TBSA, mucous membrane involvement, time from the beginning of the illness to admission, albumin level at admission, fever at admission, a history of drug allergy, comorbidities at admission (including hypertension, diabetes mellitus, gout, active infection, active malignancy, epilepsy, chronic hepatitis, connective tissue disease, thyroid dysfunction, ulcerative colitis), SCORTEN score, the initial dosage of corticosteroid, the maximum dosage of corticosteroid, time to corticosteroid tapering, and the total dosage of corticosteroids. To address potential collinearity among covariates in the multivariable model, the correlations among the covariates were checked by Pearson correlation analysis. Here, −0.5 < Correlation coefficient (r) < 0.5 indicated no high correlation (Baca et al., 2013). Patients from the two groups were matched by their propensity score in blocks of 1:2 (Jabaley et al., 2020) and were matched with not only the nearest neighbors but also the cases with identical propensity scores from the other group. The standardized mean difference (SMD) between selected variable < 0.10 was considered to reflect a good matching between two groups for that covariate (Austin, 2011). Propensity matching, SMD and *p*-value (*p* < 0.05 was considered significant) calculating, graph drawing, histogram based on the matched propensity scores, and dot plot based on SMDs before and after propensity matching were performed using STATA 13.0 (College Station, TX, USA).

### 2.4 Statistical Analysis

All data were analyzed using STATA 13.0. Mean ± standard deviation (SD) and median [interquartile range (IQR)] were used to describe continuous variables that were compared by two-tailed t-tests and Wilcoxon rank sum tests, and frequencies (percentages) were applied to describe categorical data that were compared using chi-square test, corrected chi-square test, and Fisher’s exact probability test. Standardized mortality ratios (SMRs) were calculated to compare between the observed mortalities and expected mortalities as predicted by SCORTEN (Liddell, 1984), and the 95% confidence interval (CI) of the SMR was calculated using Fisher’s exact test. A two-tailed *p* < 0.05 was considered significant. Linear and logistic regressions were used to examine the effect of IVIg on the outcomes of interest; *p* < 0.05 was considered significant.

## 3 Results

### 3.1 Patient Characteristics of the Total Sample

Baseline demographic information for the complete cohort of patients is shown in Table 1. There was no significant difference between age (*p =* 0.819), gender (*p =* 0.082), the history of drug allergy (18/163 vs. 7/61, *p* = 0.927), and comorbidities at admission (108/163 vs. 37/61, *p* = 0.435) between IVIg- and non-IVIg-treated patients, including active infection (18/163 vs. 4/61, *p* = 0.315), active malignancy (7/163 vs. 5/61, *p* > 0.250), and a variety of diseases involving respiratory, cardiovascular, digestive, urinary, hematological, and endocrine systems, metabolism and immunity disease, as well as neuropsychiatric diseases. SJS/TEN was most frequently attributed to antibiotics in the IVIg-treated group (51/163, 31.3%), followed by anticonvulsants (33/163, 20.2%), non-steroidal anti-inflammatory drugs (17/163, 10.4%), and allopurinol (18/163, 11.0%). Anticonvulsants were considered the most common causative agents in the non-IVIg-treated group (21/61, 34.4%), followed by antibiotics (14/61, 23.0%), non-steroidal anti-inflammatory drugs (10/61, 16.4%), and allopurinol (3/61, 4.9%). Moreover, 29 patients among IVIg-treated patients (17.8%) and 7 patients among non-IVIg-treated patients (11.5%) developed SJS/TEN after concurrent use of multiple drugs.

**TABLE 1:** Patient characteristics.

		Systemic corticosteroids combined with IVIg treated (*n* = 163)	Systemic corticosteroids treated (*n* = 61)	*p*
Age (years)		47.99 ± 18.81	48.67 ± 17.45	0.819
Gender				0.082
Male		83 (50.9%)	39 (63.9%)	
Female		80 (49.1%)	22 (36.1%)	
Total body surface area (%)		38.76 ± 41.42	8.68 ± 5.32	0.000
SJS		93 (57.1%)	54 (88.5%)	0.000
SJS/TEN overlap		14 (8.6%)	7 (11.5%)	0.509
TEN		56 (34.3%)	0	0.000
Mucous membrane involvement		122 (74.8%)	37 (60.7%)	0.004
	Oral and lip mucosa	118 (72.4%)	26 (42.6%)	0.000
	Ocular mucosa	84 (51.5%)	19 (31.1%)	0.006
	Genital mucosa	96 (58.9%)	24 (39.3%)	0.009
	All three sites	78 (47.9%)	15 (24.6%)	0.002
Time from the beginning of the illness to admission (days)		5.17 ± 2.39	6.05 ± 8.19	0.771
Hypoalbuminemia at admission		139 (85.3%)	37 (60.7%)	0.000
Albumin level at admission (g/L)		33.32 ± 5.42	37.28 ± 6.5	0.000
Fever at admission		116 (71.2%)	31 (50.8%)	0.004
Causative agent				
	Antibiotics	51 (31.3%)	14 (23.0%)	0.221
	Anticonvulsants	33 (20.2%)	21 (34.4%)	0.027
	Non-steroidal anti-inflammatory drugs	17 (10.4%)	10 (16.4%)	0.222
	Allopurinol	18 (11.0%)	3 (4.9%)	0.162
	Traditional Chinese medicine	9 (5.5%)	2 (3.2%)	>0.500
	Other drugs	4 (2.5%), including 1 cisplatin, 1 hydroxychloroquine, 1 fexofenadine, and 1 anesthetic analgesics	3 (4.9%), including 1 cisplatin, 1 thalidomide, and 1 gabapentin	
	Concurrent use of multiple drugs	29 (17.8%)	7 (11.5%)	0.252
	Unknown	2 (1.2%)	1 (1.6%)	1.000
A history of drug allergy		18 (11.0%)	7 (11.5%)	0.927
Comorbidities at admission		108 (66.3%)	37 (60.7%)	0.435
	Active infection	18 (11.0%)	4 (6.6%)	0.315
	Active malignancy	7 (4.3%)	5 (8.2%)	>0.250
	Respiratory diseases			
	Chronic obstructive pulmonary disease	4 (2.5%)	0	>0.500
	Bronchiectasis	2 (1.2%)	1 (1.6%)	0.617
	Cardiovascular diseases			
	Hypertension	54 (33.1%)	13 (21.3%)	0.086
	Arrhythmia	8 (4.9%)	0	>0.500
	Coronary artery disease	4 (2.5%)	0	>0.500
	Chronic heart dysfunction	3 (1.8%)	0	0.383
	Digestive diseases			
	Chronic hepatitis	4 (2.5%)	3 (4.9%)	>0.500
	Acute liver failure	2 (1.2%)	0	1.000
	Chronic liver dysfunction	2 (1.2%)	0	1.000
	Ulcerative colitis	2 (1.2%)	1 (1.6%)	1.000
	Peptic ulcer	2 (1.2%)	0	1.000
	Urinary diseases			
	Chronic kidney disease	9 (5.5%)	0	>0.100
	Hematological diseases			
	Chronic anemia	3 (1.8%)	0	0.383
	Endocrine diseases			
	Diabetes mellitus	17 (10.4%)	7 (11.5%)	0.822
	Thyroid dysfunction	3 (1.8%)	1 (1.6%)	>0.500
	Metabolism and immunity disease			
	Gout	11 (6.7%)	3 (4.9%)	>0.750
	Connective tissue disease	4 (2.5%)	1 (1.6%)	>0.900
	Neuropsychiatric diseases			
	Epilepsy	6 (3.7%)	2 (3.2%)	>0.750
	Mood disorder	3 (1.8%)	0	0.564
CORTEN		1.34 ± 1.03	0.96 ± 0.78	0.017
	0	34 (20.9%)	17 (27.9%)	0.265
	1	65 (39.9%)	31 (50.8%)	0.141
	2	47 (28.8%)	11 (18.0%)	0.100
	3	10 (6.1%)	2 (3.2%)	>0.500
	4	6 (3.7%)	0	>0.250
	5	1 (0.5%)	0	1.000
The initial dosage of corticosteroid (methylprednisolone mg/kg/day)		1.26 ± 0.3	1.21 ± 0.27	0.189
The maximum dosage of corticosteroid (methylprednisolone mg/kg/day)		1.36 ± 0.32	1.27 ± 0.24	0.001
Time to corticosteroid tapering (days)		8.76 ± 3.57	7.35 ± 3.56	0.001
The total dosage of corticosteroid (methylprednisolone mg/kg)		12 ± 6.66	9.41 ± 4.54	0.000

Combination therapeutic group contained more TEN patients (56/163 vs. 0/61, *p =* 0.000) and less SJS patients (93/163 vs. 54/61, *p* = 0.000) and had significant presentations featured by higher TBSA (38.76% ± 41.42% vs. 8.68% ± 5.32%, *p* = 0.000) and SCORTEN score (1.34 ± 1.03 vs. 0.96 ± 0.78, *p* = 0.017) compared with monotherapeutic group. IVIg-treated patients had a higher incidence of mucous membrane involvement (122/163 vs. 32/61, *p* = 0.004), including oral and lip mucosa (118/163 vs. 26/61, *p* = 0.000), ocular mucosa (84/163 vs. 19/61, *p* = 0.006), genital mucosa (96/163 vs. 24/61, *p* = 0.009), and all three sites (78/163 vs. 15/61, *p* = 0.002). Lower albumin level (33.32 ± 5.42 g/L vs. 37.28 ± 6.5 g/L, *p =* 0.000) and more common fever at admission (116/163 vs. 31/61, *p* = 0.004) were seen in patients who were IVIg treated. The distribution of SCORTEN score was different between IVIg- and non-IVIg-treated patients. The percentage of SCORTEN score of 0–1 was higher (99/163 vs. 48/61, *p* = 0.012) and the SCORTEN score of 2–5 was lower in non-IVIg-treated patients (64/163 vs. 13/61, *p* = 0.012). Except in the initial dosage of corticosteroid (mg/kg/day) (1.26 ± 0.3 vs. 1.21 ± 0.27, *p* > 0.05), there were significant differences in the maximum dosage of corticosteroid (mg/kg/day) (1.36 ± 0.32 vs. 1.27 ± 0.24, *p* = 0.001), time to corticosteroid tapering (days) (8.76 ± 3.57 vs. 7.35 ± 3.56, *p* = 0.001), and the total dosage of corticosteroid (mg/kg) (12 ± 6.66 vs. 9.41 ± 4.54, *p* = 0.000) between IVIg- and non-IVIg-treated patients. These indicated that the conditions of IVIg-treated patients were generally more serious than those of non-IVIg-treated patients. In addition to the use of IVIg, corticosteroid dose administration also differed between the 2 groups.

### 3.2 Patient Characteristics of the Propensity-Matched Sets

**FIGURE 1:**
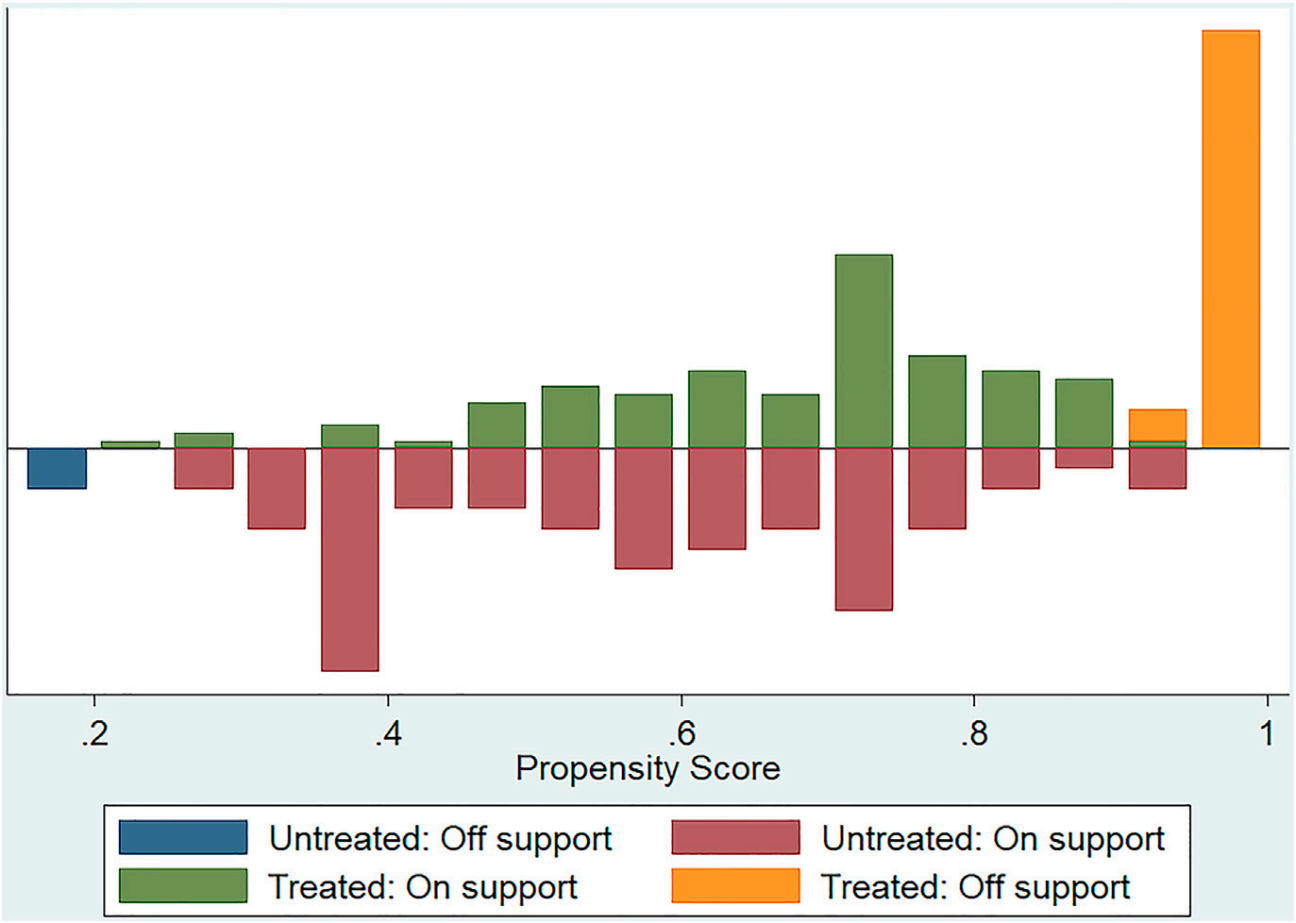
Histogram of propensity scores in 224 patients. “Treated” means patients treated with combination therapy. “Untreated” means patients treated with corticosteroids alone. “On support” indicates excellent matching. “Off support” indicates unsuccessful matching.

**FIGURE 2:**
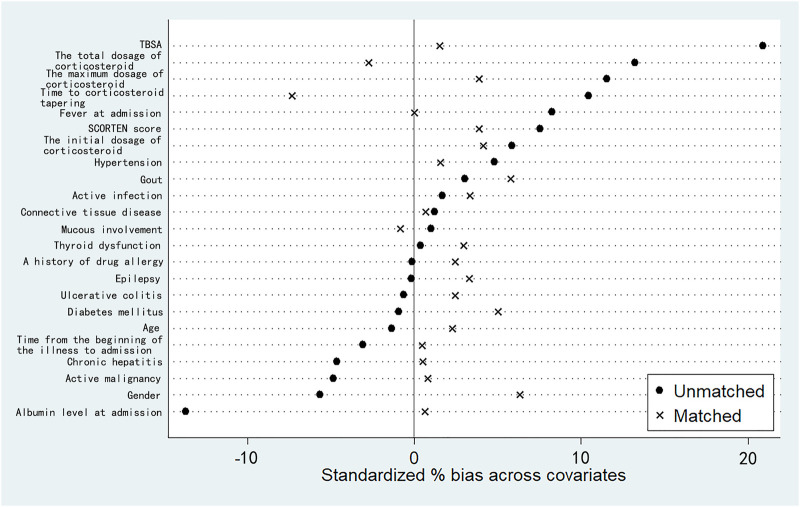
Standardized mean differences (SMDs) before and after propensity matching based on 23 independent variables. “Unmatched” represents SMDs before our propensity matching. “Matched” represents SMDs after our propensity matching. For each covariate, SMD <10% reflects a good matching.

In order to control the effect of potential confounding factors and corticosteroid treatment, we undertook a propensity score analysis to match patients between the 2 groups; a total of 145 patients were yielded in PSM, including 93 patients treated with IVIg and 52 patients not treated with IVIg. Among them, 70 patients in the combination therapeutic group and 9 patients in the monotherapeutic group were excluded from our analysis (Figure 1). SMDs before and after propensity matching are shown in Figure 2. We can see that after propensity matching, SMDs of all the variables between patients treated with/without IVIg were within 10%. SMDs for each variable after propensity matching are shown in Table 2. All the variables reflected good matching between patients treated with/without IVIg, and no significant difference was observed (Table 2), including age (*p* = 0.712), gender (*p* = 0.282), TBSA (*p* = 0.330), mucous membrane involvement (*p* = 0.118), time from the beginning of the illness to admission (*p* = 0.257), albumin level at admission (*p* = 0.147), fever at admission (*p* = 1.000), a history of drug allergy (*p* = 0.062), comorbidities at admission such as active infection (*p* > 0.750), active malignancy (*p* > 0.250), epilepsy (*p* > 0.500), chronic hepatitis (*p* > 0.500), connective tissue disease (*p* > 0.900), thyroid dysfunction (*p* = 1.000), ulcerative colitis (*p* > 0.500), SCORTEN score (*p* = 0.641), the initial dosage of corticosteroid (*p* = 0.462), the maximum dosage of corticosteroid (*p* = 0.472), time to corticosteroid tapering (*p* = 0.205), and the total dosage of corticosteroid (*p* = 0.385). Notably, some of the comorbidities at admission, such as chronic kidney disease, arrhythmia, coronary artery disease, chronic obstructive pulmonary disease, bronchiectasis, chronic anemia, chronic heart dysfunction, mood disorder, acute liver failure, chronic liver dysfunction, and peptic ulcer could not be analyzed as variables due to a lack of data in the monotherapeutic group; they were omitted in our propensity matching procedure. This implies that most of the confounding factors are balanced, both groups have comparable severity of illness, and the effects of corticosteroids could be negligible between the two groups in the present study. Moreover, all of the 23 covariates that we included in PSM had correlations of r < |0.5| with each other, and the largest coefficient r values were 0.470 (between the time to corticosteroid tapering and the total dosage of corticosteroids) and 0.457 (between the initial dosage of corticosteroid and the maximum dosage of corticosteroid). The |r| values between other covariates were all <0.3; most of them were <0.1. In addition, we compared the years of admission of the two groups not only before but also after propensity matching and concluded that there was no significant difference in admission years between the two groups (*p* = 0.416, *p* = 0.542, respectively). It can be considered that patients with combination therapy were not more prevalent in more recent years and monotherapy in earlier years.

**TABLE 2:** Propensity-matched patient characteristics.

		Systemic corticosteroids combined with IVIg treated (*n* = 93)	Systemic corticosteroids treated (*n* = 52)	Absolute standardized mean difference (SMD)	*p*
Age (years)		46.85 ± 19.11	47.28 ± 16.84	0.063	0.712
Gender				0.011	0.282
Male		45 (48.4%)	30 (57.7%)		
Female		48 (51.6%)	22 (42.3%)		
Total body surface area (%)		10.40 ± 8.20	8.86 ± 5.41	0.007	0.330
SJS		68 (73.1%)	45 (86.5%)		0.062
SJS/TEN overlap		14 (15.1%)	7 (13.5%)		0.794
TEN		11 (11.8%)	0		0.010
Mucous membrane involvement		58 (62.4%)	36 (69.2%)	0.048	0.118
Time from the beginning of the illness to admission (days)		5.15 ± 2.33	5.16 ± 1.25	0.001	0.257
Albumin level at admission (g/L)		34.43 ± 5.34	36.87 ± 6.19	0.004	0.147
Fever at admission		59 (63.4%)	28 (53.8%)	0.000	1.000
A history of drug allergy		9 (9.7%)	7 (13.5%)	0.062	0.062
Comorbidities at admission					
	Hypertension	28 (30.1%)	11 (21.2%)	0.027	0.394
	Diabetes mellitus	7 (7.5%)	5 (9.6%)	0.034	>0.900
	Active infection	7 (7.5%)	4 (7.7%)	0.014	>0.750
	Gout	6 (6.5%)	3 (5.8%)	0.005	>0.750
	Active malignancy	2 (2.2%)	3 (5.8%)	0.004	>0.250
	Connective tissue disease	3 (3.2%)	1 (1.9%)	0.021	>0.900
	Ulcerative colitis	2 (2.2%)	1 (1.9%)	0.034	>0.500
	Epilepsy	1 (1.1%)	2 (3.8%)	0.015	>0.500
	Chronic hepatitis	1 (1.1%)	2 (3.8%)	0.006	>0.500
	Thyroid dysfunction	1 (1.1%)	1 (1.9%)	0.009	1.000
SCORTEN		0.91 ± 0.83	0.90 ± 0.7	0.013	0.641
	0	31 (33.3%)	16 (30.8%)		
	1	43 (46.2%)	26 (50%)		
	2	16 (17.2%)	9 (17.3%)		
	3	2 (2.2%)	1 (1.9%)		
	4	1 (1.1%)	0		
The initial dosage of corticosteroid (methylprednisolone mg/kg/day)		1.23 ± 0.25	1.21 ± 0.24	0.021	0.462
The maximum dosage of corticosteroid (methylprednisolone mg/kg/day)		1.28 ± 0.22	1.25 ± 0.24	0.019	0.472
Time to corticosteroid tapering (days)		7.61 ± 3.64	7.95 ± 3.12	0.039	0.205
The total dosage of corticosteroid (methylprednisolone mg/kg)		9.70 ± 4.60	9.84 ± 3.91	0.020	0.385

### 3.3 The Clinical Outcomes of Propensity-Matched Patients

We then compared the expected mortality and actual mortality in patients treated with/without IVIg. As shown in Table 3, although there was no significant difference between the totally predicted and actual mortality (5.271, 5.7% vs. 2, 2.2%, *p* > 0.250), the actual mortality was lower than it was predicted in patients treated with IVIg, the SMR was 0.38 (95% CI, 0.00–0.91), which suggested that the mortality rate was approximately 62% lower for patients treated with combination therapy than those treated with routine therapy. As shown in Table 4, there was no significant difference between the totally predicted and actual mortality in non-IVIg-treated group (2.628, 5.1% vs. 2, 3.8%, *p* = 1.000), the SMR was 0.75 (95% CI 0.00–1.76), which suggested that mortality rate was 25% lower for patients treated with corticosteroids than those treated with routine therapy.

**TABLE 3:** Mortality data of propensity-matched SJS/TEN patients treated with combination therapy.

SCORTEN	n	Expected mortality, %	Expected mortality	Actual Mortality, %	Actual Mortality	*p*	Standardized mortality ratio (95% CI)
0	31	1.2	0.372	0	0	1.000	0
1	43	3.9	1.677	2.3	1	1.000	0.59 (0.00–1.74)
2	16	12.2	1.952	6.3	1	1.000	0.52 (0.00–1.49)
3	2	32.4	0.648	0	0	1.000	0
4	1	62.2	0.622	0	0	1.000	0
Total	93	5.7	5.271	2.2	2	>0.250	0.38 (0.00–0.91)

**TABLE 4:** Mortality data of propensity-matched SJS/TEN patients treated with systemic corticosteroids.

SCORTEN	*n*	Expected mortality, %	Expected mortality	Actual Mortality, %	Actual Mortality	*p*	Standardized mortality ratio (95% CI)
0	16	1.2	0.192	0	0	1.000	0
1	26	3.9	1.014	3.8	1	1.000	0.97 (0.00–2.86)
2	9	12.2	1.098	11	1	1.000	0.90 (0.00–2.58)
3	1	32.4	0.324	0	0	1.000	0
Total	52	5.1	2.628	3.8	2	1.000	0.75 (0.00–1.76)

As shown in Table 5, there were 2 patients each who died in both groups; combination therapy for patients seemed to display a tendency to decrease the mortality rate when compared with corticosteroid therapy alone, but the difference was not significant (2.2% vs. 3.8%, *p* > 0.900). Combination therapy also appeared to reduce the time to the arrest of progression (3.88 ± 1.55 vs. 5.44 ± 1.19, *p* = 0.000) and the total hospitalization time (10.95 ± 3.78 vs. 14.32 ± 4.89, *p* = 0.000) significantly. We analyzed the nosocomial infection rate of patients treated with/without IVIg. In the combination therapeutic group, 6 patients suffered from nosocomial infection, 2 patients (2.2%) had lower respiratory tract infection (2 cases’ sputum cultures grew *Staphylococcus aureus*), 2 (2.2%) had urinary tract infection (Gram-negative bacilli >10^5^ cfu/ml in the clean midstream urine of 2 cases), 1 (1.1%) had skin infection (*Candida albicans* was identified from wound culture), and 1 (1.1%) had digestive tract infection (oral swab cultured *C. albicans*). In the corticosteroid therapeutic group, there were 9 patients who experienced nosocomial infection, including 3 cases (5.8%) of skin infection (2 infected by *S. aureus*, 1 infected by *Enterococcus faecalis*), 2 cases (3.8%) of lower respiratory tract infection (1 infected by *Klebsiella pneumoniae*, 1 infected by *Aspergillus*), 2 cases (3.8%) of virus infection (1 infected by Epstein–Barr virus, 1 infected by *Cytomegalovirus*, positive viral DNA or positive antiviral IgM was detected from patient’s blood), 1 case (1.9%) of digestive tract infection (*Clostridium difficile* was cultured from the stool), and 1 case (1.9%) of urinary tract infection (Gram-negative bacilli). The overall infection rate in the combination therapeutic group was significantly lower than that in the corticosteroid therapeutic group (6/93, 6.5% vs. 9/52, 17.3%, *p* = 0.040); similarly, the incidence of skin infections was significantly lower in the combined therapy group (1/93, 1.1% vs. 3/52. 5.8%, *p* < 0.025). The prevalence of infection did not differ significantly among the other field sites (all *p*-values were >0.05).

**TABLE 5:** Clinical outcomes of propensity-matched SJS/TEN patients treated with systemic corticosteroids or combination therapy.

		Systemic corticosteroids combined with IVIg treated (*n* = 93)	Systemic corticosteroids treated (*n* = 52)	*p*
Mortality		2 (2.2%)	2 (3.8%)	>0.900
Time to arrest of progression(d)		3.88 ± 1.55	5.44 ± 1.19	0.000
Total hospitalization time(d)		10.95 ± 3.78	14.32 ± 4.89	0.000
Infection		6 (6.5%)	9 (17.3%)	0.040
	Lower respiratory tract	2 (2.2%)	2 (3.8%)	>0.900
	Skin	1 (1.1%)	3 (5.8%)	<0.025
	Digestive tract	1 (1.1%)	1 (1.9%)	>0.500
	Urinary tract	2 (2.2%)	1 (1.9%)	>0.500
	Viruses	0	2 (3.8%)	0.127

In our analysis of logistic and linear regressions (Table 6), IVIg tended toward reducing the time to arrest of progression by 1.56 days (*p* = 0.000) and the length of hospital stay by 3.37 days (*p* = 0.000). The mortality rate for patients treated with IVIg combined with corticosteroids was 45% lower than those only treated with corticosteroids therapy, although it was not statistically significant [odds ratio (OR) = 0.55, 95% CI 0.07–4.02, *p* = 0.555]. The total infection rate of patients treated with combination therapy tended to decrease by 67% compared to patients treated with corticosteroids alone (OR = 0.33, 95% CI 0.11–0.99, *p* = 0.047).

**TABLE 6:** Multivariate regression of IVIg on outcomes.

Outcome	Effect estimate (Std. Error)	OR (95% CI)	*p*
Mortality	−0.60 (1.02)	0.55 (0.07–4.02)	0.555
Time to arrest of progression (days)	−1.56 (0.23)		0.000
The lengths of hospital stay (days)	−3.37 (0.78)		0.000
Infection	−1.11 (0.56)	0.33 (0.11–0.99)	0.047

## 4 Discussion

The main findings of our propensity-matched retrospective investigation are that the actual mortality rates of patients treated with corticosteroids alone or IVIg combined with corticosteroids tend to be lower than those predicted by SCORTEN, although there was no significance. Compared with those treated by corticosteroids alone, combination therapy was prone to reducing mortality by 45% (*p* = 0.555) and infection rate by 67% (*p* = 0.047), especially reducing the incidence of skin infection (*p* < 0.025) in SJS/TEN patients and the time to arrest of progression by 1.56 days (*p* = 0.000) as well as the length of hospital stay by 3.37 days (*p* = 0.000). These findings are in agreement with our previous observations (*L* Yang et al., 2020; *Y* Yang et al., 2009). Observational studies have some advantages compared with randomized controlled trials (RCTs), such as cost saving; the settings may be considered to reflect the true state of how the treatment is administered in clinical practice and the ability to explore interventions or exposures when assigning subjects randomly would be unethical. However, the disadvantages are also obvious. The main disadvantage of observational data is the vulnerability to effects of confounding; all baseline characteristics are considered potential confounding factors when there are systematic differences in baseline characteristics between treatment groups, which may lead to misleading conclusions because of the effect of systematic differences in baseline characteristics between treated and control subjects, but not the effect of treatment (Austin et al., 2018). In our study, we selected 23 independent variables that may roughly reflect the baseline status of SJS/TEN patients and conducted a PSM analysis between patients treated with IVIg combined with corticosteroids and patients treated with corticosteroids alone to ensure the overall demographic characteristics, illness severity, and treatments other than IVIg were similar between the two groups, which may provide more convincing information and reference value for IVIg in clinical treatment of SJS/TEN. In addition, we selected a ratio of 1:2 for matching as bias due to matching of increasingly dissimilar subjects may be introduced in larger ratios, although the standard error of the estimate is found to be decreased (Austin, 2010).

Since its publication in 2000 (Bastuji-Garin et al., 2000), the SCORTEN score has been acknowledged and subsequently validated. Although the accuracy has been doubted (von Wild et al., 2012; Zavala et al., 2018) and some researchers suggested a redefinition of the scale for several factors such as comorbidities (e.g., renal impairment) (Hung et al., 2009; Noe et al., 2019), involved body surface area (Bansal et al., 2015), and patient’s age (Sekula et al., 2011) and gender (Hsu et al., 2016; Papp et al., 2018) deserve some considerations, the results of the latest meta-analysis indicated there were no significant differences between the actual mortality and the one predicted by SCORTEN (Torres-Navarro et al., 2020a).

Of the two patients who died in our combination therapy-treated cohort, one had a SCORTEN of 1, 9% TBSA involvement, had underlying hypertension, and died of pneumonia. The other had a SCORTEN of 2, 6% TBSA involvement, had underlying heart valve replacement, and passed from basilar artery apex infarction and cerebral hemorrhage. One of the dead patients in the corticosteroid-treated group had a SCORTEN of 1, 9% TBSA involvement, had underlying acute pityriasis licheniformis, and died of septic shock. The other had a SCORTEN of 2, 7% TBSA involvement, had underlying diabetes mellitus, and passed from multiple organ dysfunction syndrome (MODS). Among our total 224 patients, we actually observed 9 deaths, while the mortality predicted by SCORTEN was 19.90 deaths (*p* = 0.035). Although the actual mortality rates were prone to be less than they were predicted in both of our groups and patients treated by IVIg combined with corticosteroids seemed to have a 45% lower mortality rate than those treated with corticosteroid therapy alone, the ability of IVIg and corticosteroids to reduce mortality in SJS/TEN patients remains controversial.

The usage of corticosteroids is concerning for increasing the risk of protein catabolism and bacterial infection/sepsis and slowing the rate of epithelialization (McCullough et al., 2017; Tocco-Tussardi et al., 2017). Early and recent observational studies suggested significantly higher rates of infection, complications, and increased mortality (Abou-Taleb et al., 2020; Halebian et al., 1986; Kelemen et al., 1995; Schneck et al., 2008); an updated mortality analysis of the RegiSCAR study and a systemic review of case series based on SCORTEN algorithm failed to confirm a survival benefit for systemic corticosteroid-treated patients (Roujeau and Bastuji-Garin, 2011; Sekula et al., 2013). However, there still have been many research (Kim et al., 2012; Liu et al., 2016; Schneck et al., 2008) and systemic reviews (Zimmermann et al., 2017) supporting that systemic corticosteroids were life-saving and could reduce ocular complications. In the present study, there were 2 deaths in SJS/TEN patients treated by systemic corticosteroids without IVIg from 2008 to 2019, and the actual mortality was lower than the mortality predicted by SCORTEN (2/61, 3.3% vs. 3.403/61, 5.6%, *p* > 0.05), although it was not significant. However, systemic corticosteroids should be used cautiously and correctly because of the adverse effects; our previous research revealed that the use of high-dose corticosteroid may bring complications associated with it, consisting of infection, hyperglycemia, electrolyte disturbance, gastrointestinal hemorrhage, and hypertension. The time to corticosteroid tapering (≥12 days) and the maximum dosage of corticosteroid (≥1.5 mg/kg/day) were defined as two of the most relevant factors of SJS/TEN patients’ infection (L Yang et al., 2020). Based on our many years of treatment experience, once the condition of the patient was under control (Nikolsky sign turning negative, no new eruptions, and exudation improved) and reepithelialization had started, we always taper the dose of corticosteroid promptly.

IVIg is one of the most frequently applied therapies for SJS/TEN. However, the usage of IVIg remains controversial, whether IVIg is beneficial or not? If it is, low-dose or high-dose regimen should be used? These questions have always troubled us. The application of IVIg started based on the finding that Fas-mediated keratinocyte apoptosis could be inhibited by human IVIg products *in vitro*, and no mortality was observed in a pilot study that administered IVIg to 10 TEN patients (Viard et al., 1998). While some retrospective studies have concluded that IVIg treatment is effective in SJS/TEN (Metry et al., 2003; Morici et al., 2000; Prins et al., 2003a; Prins et al., 2003b; Trent et al., 2003), and recent meta-analyses have shown that high-dose IVIg (≥2 g/kg) has a beneficial effect in decreasing the mortality of SJS/TEN (Barron et al., 2015), a retrospective study of IVIg treatment suggested that IVIg did not significantly improve survival both at high (≥3 g/kg) and low (<3 g/kg) dosages (Lee et al., 2013). Furthermore, the latest meta-analyses and systematic reviews have demonstrated that IVIg was not associated with a promising survival benefit (Torres-Navarro et al., 2020b; Zimmermann et al., 2017). However, plenty of studies indicated that IVIg combined with corticosteroids provides a survival benefit. Low-dose (0.2–0.5 g/kg) IVIg combined with corticosteroids showed reduced recovery time and mortality in TEN patients (Jagadeesan et al., 2013); a recent retrospective analysis concluded that IVIg combined with corticosteroid had the lowest SMR (Micheletti et al., 2018), and recent systematic reviews and meta-analyses showed that using IVIg plus corticosteroids led to less deaths than that predicted by SCORTEN (Torres-Navarro et al., 2020b; 2021). The latest systematic review and meta-analysis confirmed that corticosteroids and IVIg combination therapy was the only treatment with significant survival benefits (SMR, 0.53; 95% CI 0.31–0.93) (Tsai et al., 2021). Our previous study suggested that compared with solo administration of corticosteroids, IVIg (2 g/kg) combined with corticosteroids led to a reduction of mortality and the times of arrested progression and hospitalization in SJS/TEN patients (Y Yang et al., 2009). In the present study, our results further supported the conclusions previously confirmed.

In addition, we identified that patients treated with combination therapy were less likely to have secondary infections than patients treated with only corticosteroids at similar doses. This suggests that IVIg may have an ability to enhance resistance to infections, leading to a decrease of mortality rate and total hospitalization time. It is also worth mentioning that the application of IVIg may lead to some adverse reactions such as chills, fever, nausea, rashes, and chest tightness; the involvement of blood system (hemolysis, thrombosis), nervous system (headache, aseptic meningitis), and urinary system (transient increase of urea nitrogen and serum creatinine); and other adverse reactions (alopecia, viral infection, uveitis, etc.) (Späth et al., 2015). Although in our department, only chest tightness and shivering have been observed occasionally and did not affect subsequent application.

A variety of treatments for SJS/TEN have been reported and have always been controversial, apart from supportive care. In recent years, supportive care has received a great deal of attention; early recognition and immediate withdrawal of any potential causative drugs are essential for reducing fatality in SJS/TEN (Lerch et al., 2018; Schneider and Cohen, 2017). Immediate transfer to a specialized unit, for example, intensive care unit or burn center, plays a significant role in supportive care (McCullough et al., 2017; Micheletti et al., 2018). Most of the SJS/TEN patients were admitted to the intensive care unit of our department with reverse-isolation procedures for better skin care. Optimal local wound care is critical, and silver-releasing non-adhesive wraps/dressings are regarded as a preferred treatment, although there have not been studies comparing them and other local wound dressings such as biosynthetic skin substitutes. In our department, oozing areas are covered by silver-releasing nonstick dressings impregnated with isotonic sodium chloride solution (normal saline) and changed at appropriate times based on local conditions; when there is no exudation, cream is also applied (Y Yang et al., 2009). In addition, we need to pay extra attention to nutritional support, fluid compensation, and electrolyte balancing. The gastrointestinal function could be affected by extensive epithelial exfoliation and the application of corticosteroids in SJS/TEN, which results in dysphagia and poor absorption. There are no published estimates of energy needs in adults. Graves et al. (2016) suggested that SJS/TEN patients required a median 24.2 kcal/kg/day (IQR, 19.4–29.9). For pediatric SJS/TEN patients, the energy requirements are estimated by the following equation: (preinjury weight (kg) × 24.6) + (wound size (% of body surface area) × 4.1) + 940 calories (Mayes et al., 2008). In our SJS/TEN patients, hypoalbuminemia at admission and electrolyte disturbance are extremely common problems. These imply that fluid compensation with albumin solution (5% human albumin, 1 ml/kg/%TBSA) and electrolyte solution (0.7 ml/kg/%TBSA) is also necessary (Shiga and Cartotto, 2010). In patients with genitourinary and ocular involvement, urologic and ophthalmologic consultations are indispensable (Schneider and Cohen, 2017).

Currently, there is strong evidence indicating that cyclosporine A (CsA) could reduce the mortality of SJS/TEN patients (Lee et al., 2017; Mohanty et al., 2017; Ng et al., 2018; Singh et al., 2013; Torres-Navarro et al., 2020b; Valeyrie-Allanore et al., 2010). The results of these studies suggest that the administration of CsA 3–5 mg/kg per day as early as possible in SJS/TEN may be beneficial and may bring better efficacy than IVIg and other therapies (González-Herrada et al., 2017; Torres-Navarro et al., 2020b; Zimmermann et al., 2017). Recently, a SCORTEN-based systematic review and meta-analysis suggested that CsA and IVIg plus corticosteroids led to less deaths than those predicted by SCORTEN (Torres-Navarro et al., 2021). Although there are some evidence that compared with supportive care, CsA does not significantly benefit the survival of SJS/TEN (Kirchhof et al., 2014; Lee et al., 2017; Sekula et al., 2013), we still think it may have a better therapeutic effect compared with the combination therapy of systemic corticosteroids and IVIg especially in children and adults with a contraindication of corticosteroids. Tumor necrosis factor-α (TNF-α) inhibitors such as etanercept and infliximab may be promising agents with therapeutic potential for SJS/TEN. The latest RCT has demonstrated that the mortality of etanercept-treated SJS/TEN patients was lower than that predicted by SCORTEN, and shorter healing time was observed compared to corticosteroid-treated patients (Wang et al., 2018). In a recent retrospective study, although patients treated with etanercept combined with IVIg had higher SCORTENs than those of patients treated by IVIg alone, no significant difference was seen in mortality between the two cohorts (Pham et al., 2019). Infliximab has also been proven to be effective in reducing the mortality compared with SCORTEN-predicted mortality (Patmanidis et al., 2012; Scott-Lang et al., 2014; Zárate-Correa et al., 2013). However, there are some studies questioning the therapeutic effect of TNF-α inhibitors; a potent TNF-α inhibitor thalidomide was reported to increase the mortality (Wolkenstein et al., 1998), and recent RCT as well as systematic review and meta-analysis suggested that etanercept was not statistically more effective than corticosteroid in SJS/TEN (Torres-Navarro et al., 2020b; Wang et al., 2018). The therapeutic effect of TNF-α antagonists is still under observation, and more data are needed to support their effectiveness.

In our department, combination therapy for SJS/TEN only consists of systemic corticosteroids and IVIg. To date, CsA or TNF-α antagonists have not been combined with either of them. Since some retrospective studies and case reports have demonstrated that etanercept combined with IVIg (Pham et al., 2019), infliximab combined with IVIg (Patmanidis et al., 2012), dexamethasone combined with CsA and etanercept (Coulombe et al., 2019) achieved a satisfactory outcome, we have been considering joint usage of them in future therapeutic approaches for SJS/TEN patients.

Our analysis presented in this paper was anticipated to provide some references for clinical treatment of SJS/TEN, especially in countries with a limited health budget. Due to the rarity and severity of SJS/TEN, an RCT is difficult to implement. Although a retrospective study could provide us with some valuable evidence, one limitation of our study is its single-center design. The heterogeneity among patients can never be fully excluded; although the administration of corticosteroids was matched between patients treated with or without IVIg, corticosteroid dosage was not standard, which can be a confounding factor in the study. In our clinical work, for TEN patients who presented with more severe disease, we preferred to apply IVIg combined with corticosteroids; this resulted in the inclusion of many TEN patients in the combined treatment group, but not in the other group, which created a probable selection bias. In the process of propensity matching, in order to ensure the comparability between the two groups of patients, it is inevitable to eliminate some TEN patients with severe conditions and SJS patients with very mild conditions, which undoubtedly affects the representativeness of our study participants and makes our conclusions not applicable for patients with severe TEN and mild SJS. Whether combined therapy can improve the prognosis better than monotherapy in these conditions remains to be investigated further. In addition, the biases in diagnosis, case selection, and unidentified or unmeasured confounders are inherent limitations of retrospective studies. The number of patients included in our study was small but as many covariates as possible were included in our propensity matching. The introduction of more covariates may cause no well-matched patients in the other group can be found for some patients, make the results difficult to reflect the real situation to some extent, and further narrow down the sample size. Although all of the 23 covariates we included in propensity matching had correlations of r < |0.5| with each other, a certain correlation probably existed among individual covariates; this will likely lead to estimation errors of the parameters. Propensity matching was conducted to restrict the impact of measured confounders; however, propensity matching cannot substitute for true randomization and other unmeasured confounders may bring a systematic selection bias. Additionally, propensity matching only evaluates the average treatment effect; this study may have limited generalizability. In the future, data collection from multicenters with larger numbers of patients is warranted for better standardization and comparison; well-designed prospective studies should be conducted to identify the standard therapy for SJS/TEN.

## Data Availability

The original contributions presented in the study are included in the article/Supplementary Material, further inquiries can be directed to the corresponding authors.
